# *M. paratuberculosis* Heat Shock Protein 65 and Human Diseases: Bridging Infection and Autoimmunity

**DOI:** 10.1155/2012/150824

**Published:** 2012-09-29

**Authors:** Coad Thomas Dow

**Affiliations:** Chippewa Valley Eye Clinic, Wisconsin and Eye Research Institute, University of Wisconsin-Madison, 2715 Damon Street, Eau Claire, WI 54701, USA

## Abstract

*Mycobacterium avium* subspecies *paratuberculosis* (MAP) is the known infectious cause of Johne's disease, an enteric inflammatory disease mostly studied in ruminant animals. MAP has also been implicated in the very similar Crohn's disease of humans as well as sarcoidosis. Recently, MAP has been associated with juvenile sarcoidosis (Blau syndrome), autoimmune diabetes, autoimmune thyroiditis, and multiple sclerosis. While it is intuitive to implicate MAP in granulomatous diseases where the microbe participates in the granuloma, it is more difficult to assign a role for MAP in diseases where autoantibodies are a primary feature. MAP may trigger autoimmune antibodies via its heat shock proteins. Mycobacterial heat shock protein 65 (HSP65) is an immunodominant protein that shares sequential and conformational elements with several human host proteins. This molecular mimicry is the proposed etiopathology by which MAP stimulates autoantibodies associated with autoimmune (type 1) diabetes, autoimmune (Hashimoto's) thyroiditis, and multiple sclerosis. This paper proposes that MAP is a source of mycobacterial HSP65 and acts as a trigger of autoimmune disease.

## 1. Introduction

The term “molecular mimicry” is accredited to Damian in 1964. He was first to suggest that antigenic elements of microorganisms may resemble antigenic elements of their host. Damian suggested that this similarity served as a defense mechanism of a microorganism from the host's immune system and prevented the development of immune response to the microorganism, thereby protecting it from host defense [1]. Over time the term “molecular mimicry” was given a different meaning, namely, antigenic elements of microorganisms may trigger an autoimmune response that harms the host. The concept of molecular mimicry is based on a structural similarity between a pathogen and self. The similarity could be expressed as shared amino acid sequences or similar conformational structure between a pathogen and self-antigens. Molecular mimicry has become a very popular explanation for the frequent association of infection with autoimmune disease [2].

Under cellular stress such as heat, ethanol, heavy metals, endotoxins, and inflammatory processes, heat shock proteins (HSPs) are rapidly synthesized, increasing their concentration in the intracellular compartment as well as on the cell surface [3–9]. Heat shock proteins are found in virtually all life forms and are closely linked to the immune response. HSP65 of microorganisms is an immunodominant antigen. In human mycobacterial infection, for example, it has been estimated that up to 40% of the T-cell response is directed against this single protein [10, 11]. Although there is great homology between mycobacterial HSP65 among the species, genotyping of the 3′ end of the HSP65 gene has proven to unambiguously distinguish between the *M. avium *complex of mycobacteria as well as the cattle and sheep strains of *M. paratuberculosis *[12]. Immune responses to these proteins have been described in both infectious [13] and autoimmune diseases [14, 15] in human and animal models, suggesting that they could be attractive target molecules for interfering with these processes [16, 17].

This paper proposes that *Mycobacterium avium *ss. *paratuberculosis *(MAP) is a source of mycobacterial HSP65 and acts as a driver of autoimmunity [18].

## 2. MAP

MAP is a Gram-positive, acid-fast staining small rod-shaped bacterium. As with members of the Mycobacteriaceae genus, it has a unique cell wall structure rich in complex lipids. The thick and chemically distinctive cell wall of mycobacteria is largely responsible for the robust nature of these bacteria, both within the host cell and in the environment. The pathogenic potential of mycobacteria is correlated with their growth rate. Paradoxically, slow-growing mycobacteria are more virulent than fast-growing mycobacteria. With the exception of *Mycobacterium leprae *(the cause of leprosy in humans), which cannot be cultured in vitro, MAP has the slowest growth rate of pathogenic mycobacteria. After isolation from infected animals and grown under optimal conditions colonies of MAP are typically not visible for 3 months or more [19]. MAP causes a chronic granulomatous inflammation of the intestines in ruminant animals called Johne's disease. Mostly studied in dairy cattle, goats, and sheep, MAP also causes a chronic inflammation of the intestines in beef cattle and in a wide variety of other domestic and wild ruminants. MAP-induced enteric inflammation has been found in monogastric animals including dogs and pigs as well as four different types of subhuman primates—macaques, baboons, gibbons, and cotton-top tamarins [20]. A majority of the dairy herds in the United States and Europe have MAP infected animals within the herd [21]. 

## 3. MAP and Human Exposure

Infected cows shed up to 1.6 × 10^7^ organisms per 2 grams of manure (0.07 oz)—a dose large enough to infect a calf. A single high-shedding animal can excrete up to 15 *gallons* of such contaminated manure per day—a staggering *25,000 infective doses per day *[22]. MAP is present in pasteurized milk [23, 24], infant formula made from pasteurized milk [25], surface water [26, 27], soil [26], cow manure “lagoons” that leach into surface water, cow manure in both solid and liquid forms that is applied as fertilizer to agricultural land [28, 29], and municipal tap water [29, 30], providing multiple routes of transmission to humans.

## 4. MAP and Human Granulomatous Disease: Crohn's and Sarcoidosis

In addition to Johne's disease of animals, MAP is the putative cause of the strikingly similar Crohn's disease of humans. Although there has been a century-long debate, the role of MAP in Crohn's is evolving from controversial to compelling [31–33]. The major source of the debate is that conventional methods of detecting bacteria—namely, culture and stain—are largely ineffective in detecting MAP. However, with newer laboratory techniques, primarily PCR, evidence of MAP is readily found in Crohn's tissues [34, 35]. In a study that evaluated the inflammatory bowel disease of attendants of goat herds where caprine Johne's disease is endemic, MAP was detected in the attendants compared to controls; the risk was correlated with the duration of association with the endemic goat herd [36]. The DNA of MAP can be identified within the granulomas of Crohn's biopsies [37] and, with extreme care and patience, MAP can be grown from the gut and blood of Crohn's patients [38–40]. In limited series, antimycobacterial therapy directed at MAP has been shown to have a favorable effect on patients with Crohn's disease [41].

Moreover, MAP has been historically linked is sarcoidosis; a multisystem inflammatory disease in which DNA evidence of MAP has been found (sporadically) in sarcoid granulomas [42]. Juvenile sarcoidosis (Blau syndrome) is an inherited granulomatous disease of children. The DNA of MAP was detected from every sample in a small series of archived tissues [43]. 

## 5. Genetics of Mycobacterial Infection and Autoimmunity

A complete discussion of shared genetic association to mycobacterial infection and autoimmune disease is beyond the scope of this paper. However, the role of the SLC11a1 gene is worth discussing in some depth. Natural resistance-associated macrophage protein 1 (NRAMP1) is now referred to as SLC11a1 (solute carrier 11a1). The gene that encodes for this protein is recognized as having a role in the susceptibility of humans and animals to a number of infections, including mycobacterial infections, and is associated with a number of autoimmune diseases as well.

The product of the SLC11a1 gene modulates the cellular environment in response to activation by intracellular pathogens by acidifying the phagosome thus killing the pathogen [44]. As such, it plays a role in host innate immunity [45]. Mutation of SLC11a1 impairs phagosome acidification yielding a permissive environment for the persistence of intracellular bacteria [46].

SLC11a1 polymorphisms are associated with paratuberculosis in cattle [47], goats [48], and sheep [49]. When researchers at the Belgium Pasteur Institute developed a murine model for MAP infection, they created an SLC11A1 defect mouse [50]. Given the pivotal roles that SLC11A1 plays in innate immunity and, as such, is not surprising that the relationship between polymorphisms in SLC11A1 and a number of mycobacterial as well as autoimmune diseases has been explored [51]. Associations have been found with leprosy [52], tuberculosis [53], and the aforementioned sarcoidosis [54]. Additionally, SLC11a1 associations have been found with rheumatoid arthritis [55], visceral leishmaniasis [56], multiple sclerosis [57, 58], inflammatory bowel disease [59–61], and type 1 diabetes mellitus (T1DM) [62, 63].

## 6. MAP and Type 1 Diabetes, Autoimmune Thyroiditis, and Multiple Sclerosis

While it is not difficult to envision a role for MAP in human disease where there is a granuloma, it is more difficult to divine a role for MAP in diseases that feature autoantibodies. This divide is bridged by the concept that MAP HSP65 mimics host protein elements. An example is that of MAP as a proposed infectious trigger of autoimmune diabetes. T1DM is an autoimmune disease manifest by progressive T cell-mediated autoimmune destruction of insulin-producing beta cells in the pancreatic islets of Langerhans [64]. In 2005, Dow postulated a causative role for MAP in the T1DM [65]. Sechi et al. in 2007 found the DNA of MAP in the blood of autoimmune (type 1) patients but not nonautoimmune (type 2) diabetics [66–68]. (Sechi also found an association of polymorphisms of the SLC11a1 gene and MAP in T1DM patients [61].) The link connecting MAP and T1DM: MAP HSP65 mimic the host pancreatic glutamic acid decarboxylase (GAD) [69]. Similar mechanisms are proposed for the role of MAP in autoimmune (Hashimoto's) thyroiditis [70, 71] and multiple sclerosis [72].

## 7. Discussion

Not specific to MAP but to mycobacteria in general, mycobacterial HSP has been found in several additional autoimmune diseases [73]: the mycobacterial HSP65 has been implicated in the pathogenesis of rheumatoid arthritis [74], autoimmune hepatitis [75], primary biliary cirrhosis [76], and scleroderma [77]. HSP65 is also implicated in multiple vasculitis-associated systemic autoimmune diseases such as Kawasaki disease [78], Behcet's disease [79], and Takayasu's arteritis [80].

Although the SLC11a1 gene was featured in our discussion, there are several gene defects that are associated with mycobacterial infection and autoimmune disease. Besides SLC11a1, genes with strong mycobacterial susceptibility/autoimmune associations are the NOD2 gene [81], VDR (vitamin D receptor) gene [82], the LTA (lymphotoxin-alpha) gene [83], and the complement C4 gene [84]. The NOD2 (CARD15) gene has been of interest as different domains of the gene are associated with two aforementioned human diseases: Crohn's and Blau syndrome [43]. 

The list of diseases in which MAP has been implicated in a causal role is growing. This paper illuminates a parsimonious path linking MAP and a number of autoimmune diseases. The link proposed is mycobacterial HSP65 of MAP.
